# The HOF structures of nitrotetraphenylethene derivatives provide new insights into the nature of AIE and a way to design mechanoluminescent materials†
†Electronic supplementary information (ESI) available: Synthetic procedures, experimental details, supplemental figures and X-ray crystallographic data for TPE2N (CCDC: 1451590), TPE3N (1451588) and TPE4N (1451589). See DOI: 10.1039/c6sc03177c



**DOI:** 10.1039/c6sc03177c

**Published:** 2016-09-02

**Authors:** Tao Yu, Depei Ou, Zhiyong Yang, Qiuyi Huang, Zhu Mao, Junru Chen, Yi Zhang, Siwei Liu, Jiarui Xu, Martin R. Bryce, Zhenguo Chi

**Affiliations:** a PCFM Lab , GDHPPC Lab , Guangdong Engineering Technology Research Center for High-performance Organic and Polymer Photoelectric Functional Films , State Key Laboratory of OEMT , School of Chemistry and Chemical Engineering , Sun Yat-sen University , Guangzhou 510275 , China . Email: yangzhy29@mail.sysu.edu.cn ; Email: ceszy@mail.sysu.edu.cn ; Email: chizhg@mail.sysu.edu.cn; b Department of Chemistry , Durham University , Durham DH1 3LE , UK

## Abstract

This study probes the effect of intramolecular rotations on aggregation-induced emission (AIE) and leads to a kind of supramolecular mechanoluminescent material. Two hydrogen-bonded organic frameworks (HOFs), namely HOFTPE3N and HOFTPE4N, have been constructed from nitro-substituted tetraphenylethene (TPE) building blocks, namely tris(4-nitrophenyl)phenylethene (TPE3N) and tetrakis(4-nitrophenyl)ethene (TPE4N). Using single-crystal X-ray diffraction analysis, two types of pores are observed in the HOFTPE4N supramolecular structure. The pore sizes are 5.855 Å × 5.855 Å (α pores) and 7.218 Å × 7.218 Å (β pores). Powder X-ray diffraction and differential scanning calorimetry studies further reveal that the α pores, which contain nitrophenyl rings, quench the emission of HOFTPE4N. This emission can be turned on by breaking the α pores in the HOFs by grinding the sample. Temperature-dependent emission studies demonstrate that the emission quenching of HOFTPE4N is attributed to the intramolecular rotations of nitro-substituted phenyl units within the space of the α pores. These results clearly reveal AIE by controlling the intramolecular rotations, which can serve as a basis for developing mechanoluminescent materials.

## Abstract 2

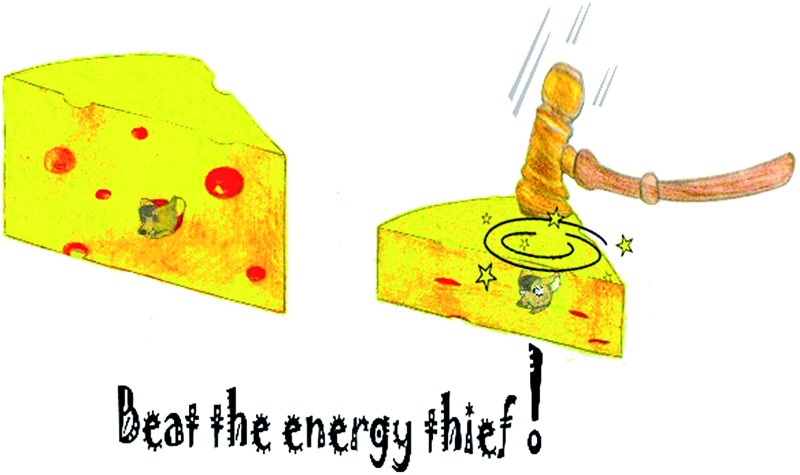
HOFs with AIE building blocks are used to reveal the nature of AIE clearly and develop a kind of supramolecular mechanoluminescent material.

## Introduction

The well-established anti-concentration quenching concept, namely aggregation-induced emission (AIE), was first reported by Tang in 2001.^1^ AIE compounds become much more emissive after aggregation because of the restriction of intramolecular rotations.^2^ With the drastic emission increase from solution states to aggregated states, these compounds are widely used as organic light-emitting diode (OLED) materials,^3^ piezochromic materials,^4^ chemosensors^5^ and biosensors.^6^ Many methods have been used to clarify the effect of intramolecular rotation on AIE phenomena,^7^ including controlling the solution viscosity and related computational calculations, but the transition to restricted rotations (“aggregation”) is still difficult to clearly demonstrate and to define and control precisely. The occurrence of restricted rotations in solution also prevents the applications of AIE materials in some ways. If the process can be induced to occur only in the solid state, especially in the crystalline state, this process will become more easily understandable and practically useful. For example, emission turn-on with well-defined restriction of intramolecular rotations could clearly provide new insights into the nature of AIE. In addition, the resulting materials could be mechanoluminescent (that is, non-emissive or weakly emissive materials become emissive under irradiation after grinding or pressing) if the restricted rotation is sensitive to an external stimulus. To achieve these aims, we now report that molecules with strong intramolecular rotations have been introduced into hydrogen-bonded organic frameworks (HOFs).

Porous organic crystalline materials (POCMs) are a research hotspot in materials science because of their widespread applications in absorption, separation, gas storage, catalysis and sensing.^8^ POCM systems include zeolites,^9^ metal–organic frameworks (MOFs),^10^ covalent organic frameworks (COFs)^11^ and HOFs.^12^ Compared with other types of organic frameworks, HOFs have outstanding features, such as solvent processability and characterization, facile regeneration by recrystallization, and self-repairing abilities.^13^ HOFs are constructed by weak noncovalent intermolecular interactions. Thus, the porous structure of HOFs may be sensitive to the external environment and to stimuli. The porosities of HOFs can provide sufficient space for intramolecular rotations and weak supramolecular interactions, which can be significantly changed in response to stimuli, *e.g.* the presence of gas or solvent guest molecules.^14^ Thus, it might be possible to tune the intramolecular rotations in HOF structures to study the nature of AIE and to develop a kind of supramolecular mechanoluminescent material.

**Fig. 1:**
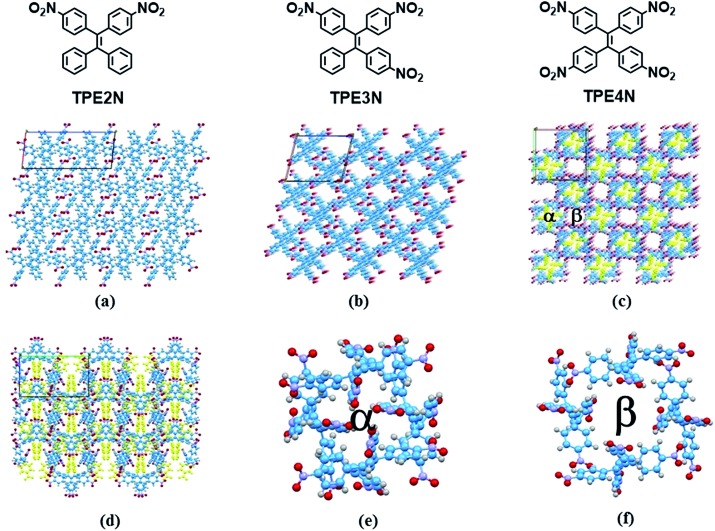
Single crystal structures of TPE2N, HOFTPE3N and HOFTPE4N ; (a) single crystal structure of TPE2N; (b) single crystal structure of HOFTPE3N viewed down the *b* axis; (c) single crystal structure of HOFTPE4N viewed down the *c* axis (the nitro substituted phenyls in pore α are labeled in yellow); (d) single crystal structure of HOFTPE4N viewed down the *b* axis (the nitro substituted phenyls in pore α are labeled in yellow); (e) structure of pore α; (f) structure of pore β.

In the present study, two HOFs are constructed using nitro-substituted tetraphenylethene building blocks to realize tunable porous structures and to reveal insights into AIE phenomena by controlling intramolecular rotations. To establish the compounds' supramolecular structures and emission properties, analyses of single crystal X-ray structures, differential scanning calorimetry (DSC), powder X-ray diffraction (pXRD) and temperature-dependent emission were carried out. Additionally, these HOF materials are shown to be mechanoluminescent materials. The building blocks TPE2N, TPE3N and TPE4N with two, three and four 4-nitrophenyl substituents, respectively (Fig. 1), were synthesized as described in Scheme S1 (ESI†). These compounds were characterized by ^1^H NMR spectroscopy and high resolution EI mass spectrometry. Light-yellow single crystals of TPE2N, TPE3N and TPE4N were obtained by recrystallization from dichloromethane–hexane mixed-solvent systems (see ESI† for detailed X-ray crystal data).

## Results and discussion

X-ray diffraction analyses reveal that single crystals based on TPE2N, TPE3N and TPE4N are in the *P*2_1_/*c*, *P*2_1_/*n* and *P*4_2_/*n* space groups, respectively. Porous HOF structures are observed for TPE3N and TPE4N, which are named HOFTPE3N and HOFTPE4N, respectively. Conversely, no porous structure is observed in TPE2N crystals. In the porous HOFTPE3N and HOFTPE4N structures, guest solvent molecules are inside the pores. The crystal structures of TPE2N, HOFTPE3N and HOFTPE4N are shown in Fig. 1. For HOFTPE3N, each TPE3N molecule is connected to 8 other TPE3N molecules with 12 weak hydrogen bonds, forming a two-dimensional layer perpendicular to the *b* axis. A three-dimensional microporous HOF structure with a pore size of 7.655 Å × 7.655 Å is obtained through further stacking of the two-dimensional layers through π–π and other supramolecular interactions along the *b* axis, as shown in Fig. 1b. Meanwhile, the HOFTPE4N supramolecular structure is formed from two-dimensional layers perpendicular to the *c* axis, which are constructed from TPE4N molecules through weak hydrogen bonds, as shown in Fig. 1c. Each TPE4N molecule is also connected to 8 other TPE4Ns with 10 weak hydrogen bonds. In HOFTPE4N, there are two types of pores with sizes of 5.855 Å × 5.855 Å (α pores) and 7.218 Å × 7.218 Å (β pores) (Fig. 1c). One nitrophenyl substituent of each TPE4N molecule is located inside the smaller α pores (marked in yellow in Fig. 1c and d). These nitrophenyl rings in the α pores, which have sufficient space to allow for intramolecular rotations, may greatly affect the emission properties of the HOFs. The detailed structures of the α and β pores of HOFTPE4N are shown in Fig. 1e and f. Subsequently, the emission “turn-on” and “turn-off” processes were investigated by destroying and reforming some building blocks in the HOF structures.

**Fig. 2:**
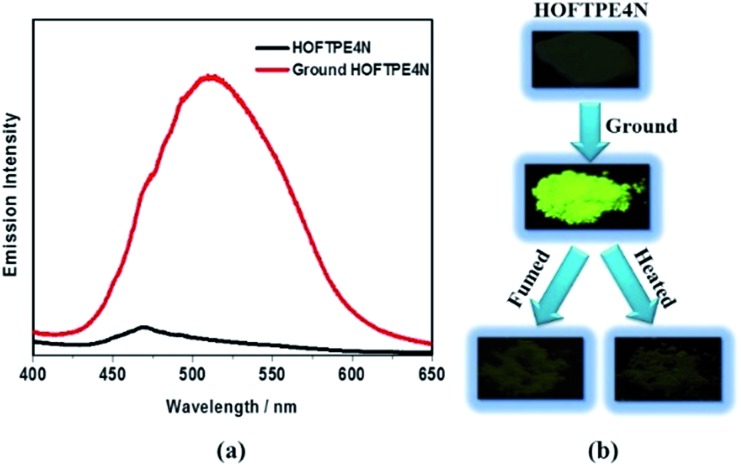
Mechanoluminescent properties of HOFTPE4N: (a) emission spectra of HOFTPE4N and ground HOFTPE4N, and (b) photographs of the mechanoluminescent properties of HOFTPE4N and the quenching processes.

Single crystals of TPE2N and HOFTPE3N are emissive with emission maxima of *λ*_max_ = 520 and 515 nm, respectively, which are attributed to π–π* transitions, according to previous reports on TPE structures.^15^ As opposed to these previous analogs, the HOFTPE4N crystals are shown to be “turn-on” mechanoluminescent materials, which has been mentioned in a Chinese patent.^5*d*^ Photographs of the turn-on and quenching processes are shown in Fig. 2. Non-emissive HOFTPE4N becomes emissive after grinding, as shown in Fig. 2b with the emergence of a new emission band at 515 nm originating from a π–π* transition. The solid-state luminescence quantum yield increases from <0.1% for HOFTPE4N to 20.8% after grinding, whereas the value for TPE4N in an amorphous state reaches 82.5%. Besides the drastic increment of emission intensity, the emission maxima of HOFTPE3N and HOFTPE4N were red-shifted after grinding which can be mainly attributed to the more planar conformation after grinding according to previous literature reports.^4*b*^ Therefore, the emission is quenched significantly by the HOF-structure formation. In addition, the emissions of ground HOFTPE4N can be further quenched by heating to 150 °C or by dichloromethane fuming. The sensitivity of HOFs to external stimuli is mainly due to their weak intermolecular interactions compared with other types of porous materials, such as MOFs and COFs.^12,14^ Comparison of the crystal structures of TPE2N, HOFTPE3N and HOFTPE4N reveals that the formation of intermolecular hydrogen bonds is not the key factor in quenching the emission band; the special structure of the α pores in HOFTPE4N, which provides sufficient space for intramolecular rotations of the nitro-substituted phenyls, caused the emission quenching in HOFTPE4N.

**Fig. 3:**
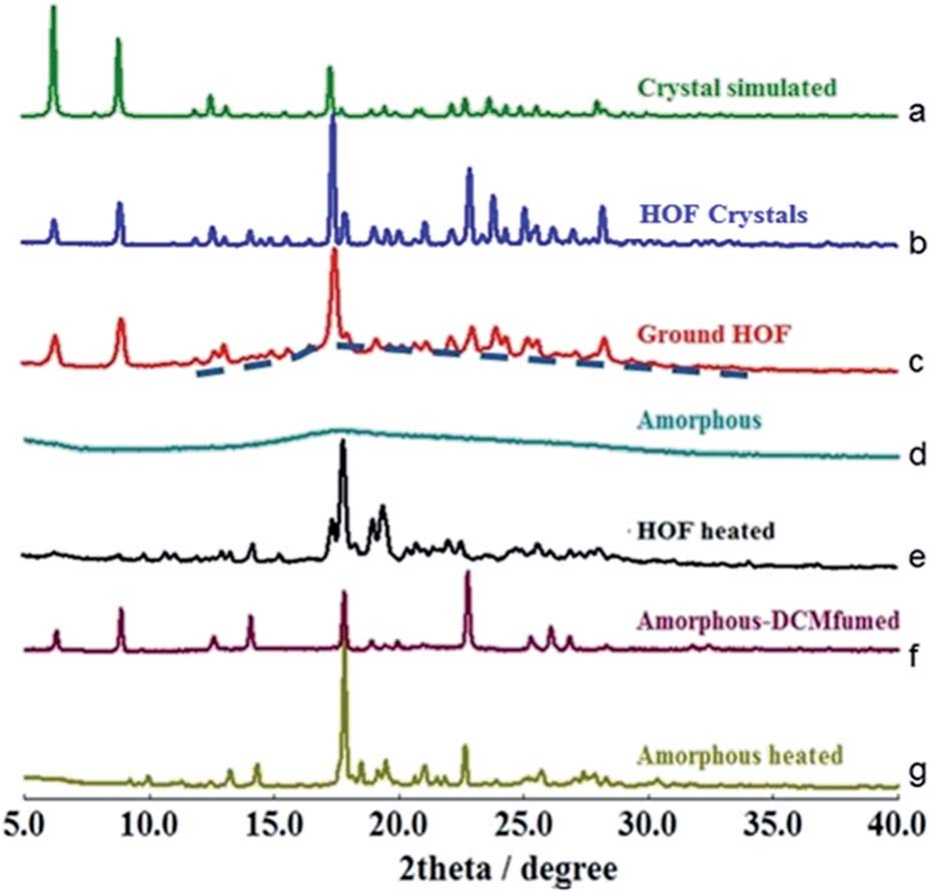
pXRD spectra of TPE4N in different states: (a) crystal simulated, (b) HOF state, (c) ground HOF state, (d) amorphous state, (e) heated HOF state, (f) dichloromethane fumed amorphous state, and (g) heated amorphous state.

To determine whether the α pore structure in HOFTPE4N is intimately related to the mechanoluminescence phenomenon, pXRD studies have been carried out on TPE4N samples in different states (HOF crystals: TPE4N in a crystalline state; ground HOF: HOF crystals were ground before measurement; amorphous: TPE4N in an amorphous state; HOF heated: the HOF crystals were heated to 200 °C for 10 minutes; amorphous-DCM fumed: amorphous TPE4N was fumed with dichloromethane for 10 hours; amorphous-heated: amorphous TPE4N was heated to 150 °C for 30 minutes). The pXRD spectra are shown in Fig. 3. In the pXRD spectra of the HOF (Fig. 3b) and the ground HOF (Fig. 3c) all the peaks become broadened and of lower intensity after grinding. Furthermore, a very broad peak at 2*θ* = 18° is observed in the ground HOF, similar to the broad peak of the amorphous state (Fig. 3d). These changes indicate that grinding has damaged the crystal structure of HOFTPE4N (both the α and β pores) to some extent. Therefore, the emission-quenching process of HOFTPE4N is mainly due to the special porous HOF structure. To further identify the structure leading to quenching, the HOFTPE4N crystals were heated to 200 °C, with the loss of guest molecules in HOFTPE4N according to the TGA curve of HOFTPE4N. The TGA data is shown in Fig. S3 (ESI†). Comparing the pXRD spectra in Fig. 3b and e, the peaks at 2*θ* = 6.5° which are consistent with the diameter of the β pores (Fig. S1†) disappeared on heating to 200 °C. This indicates that the structure of the β pores is totally destroyed by heating to 200 °C. The emission turn-on process does not occur after the destruction of the β pores, which proves that these pores are not a key factor affecting mechanoluminescence. The pXRD spectrum (Fig. 3e) indicates that TPE4N remains in a crystalline state after heating to 200 °C and that the peak at 2*θ* = 17.8° is still sharp. According to the single-crystal simulation data, 2*θ* = 17.8° is in accordance with the distance of C15 to C17 between two connected TPE4N molecules in the α pores, as shown in Fig. S2.† Structures that are similar to the α pore or part of the α pore may, therefore, still exist. In addition, the broad peak at 2*θ* = 18° in the pXRD spectrum of TPE4N in the amorphous state also indicated that the average distance between TPE4N molecules in the amorphous state is much smaller than the size of the α and β pores in the HOF state. The packing mode in the amorphous state is tighter than in the HOF state.

As aforementioned, the emission bands of ground HOFTPE4N can be further quenched by heating to 150 °C or by dichloromethane fuming. HOFTPE4N demonstrated good reversibility of the mechanoluminescence process over 10 cycles of grinding and DCM fuming with no obvious change in the emission spectra. Subsequently, the differences between these two quenching methods have been examined. TPE4N in an amorphous state was fumed with dichloromethane or heated to 150 °C. Fig. 3f shows that the pXRD spectrum of the dichloromethane-fumed sample is similar to that of HOFTPE4N crystals (Fig. 3b), whereas the pXRD spectrum of the heated sample (to 150 °C) (Fig. 3g) is similar to that of heated HOFTPE4N (Fig. 3e). These results reveal that the porous HOF structures have been reformed by dichloromethane fuming, while only structures similar to the α pore or part of the α pore have been reformed by heating to 150 °C. These results also confirm the easy reformation and self-repairing properties of the HOF structures. DSC studies show that the unique exothermic peaks at *ca.* 150 °C in the heating curves can be detected in samples of ground HOFTPE4N and amorphous-state TPE4N. However, the exothermic peak does not appear in the heating curves of HOFTPE4N heated to 150 °C. Therefore, the exothermic peaks at *ca.* 150 °C are unrelated to the formation of β pores, but are related to the recrystallization process. These results further demonstrate that heating can lead only to the reformation of structures similar to α pores or some part of the α pores, as shown in the pXRD spectrum (Fig. 3e).

**Fig. 4:**
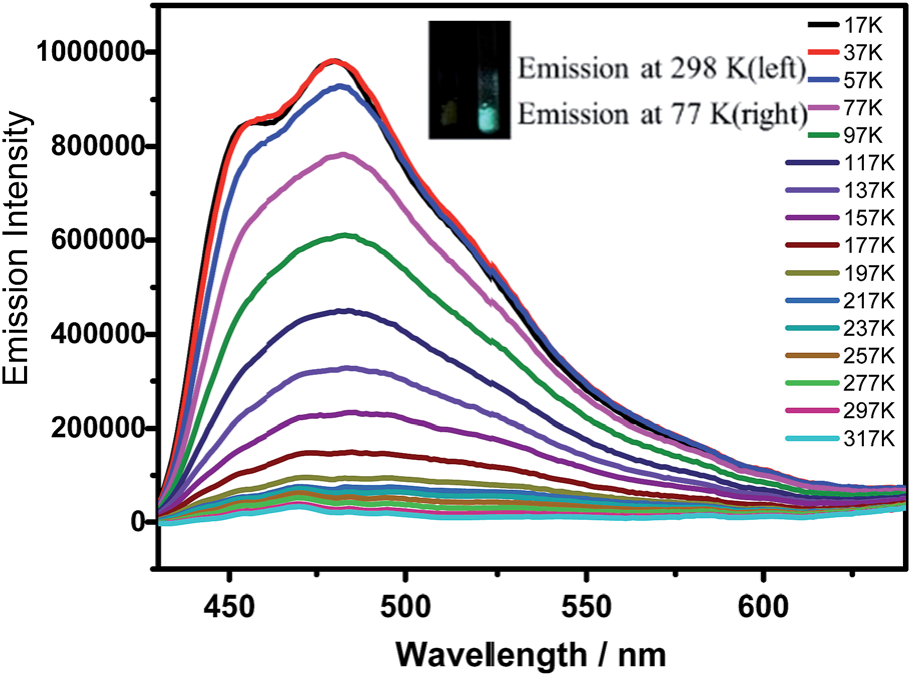
Temperature-emission spectra of HOFTPE4N. The inset shows photographs of the samples at 298 and 77 K.

Notably, α pores containing nitrophenyl groups can quench the emission of HOFTPE4N. To further explore the emission-quenching mechanism, low-temperature emission spectra have been obtained for HOFTPE4N, HOFTPE3N, and TPE2N in crystalline and amorphous states.^16^ The temperature-dependent emission spectra are shown in Fig. 4 and S7–S11.† According to the previous literature discussing the mechanism of AIE, the intramolecular rotations can be restricted with decreased temperature.^1,2,15^ For the non-emissive HOFTPE4N, emission is predicted to be turned on at low temperature if the quenching process is due to intramolecular rotations. Fig. 4 shows no observable emission band at >217 K. However, with decreasing the temperature to 217 K, emission is turned on, and the intensity drastically increased with further decreased temperatures. These results demonstrate that intramolecular rotations of the nitrophenyl groups in the α pores are the main reason for emission quenching. Thus, the mechanism of mechanoluminescence can be described as follows: the porous HOF structure (especially the α pores), which provides enough space for intramolecular rotations, quenches the emission of TPE4N. The emission is turned on/off by destroying or reforming the porous HOF structures. In addition, designing AIE molecules with a large number of intermolecular hydrogen bonds might become a new strategy for obtaining mechanoluminescent materials.

The emission bands of HOFTPE4N also show fairly well-resolved vibronic structures with vibrational progressional spacings of *ca.* 1009 cm^–1^ at low temperatures (<97 K), which are typical of *ν*(C=C) stretching modes. The emission spectra of HOFTPE3N and TPE2N in the crystal state, as well as TPE2N, TPE3N and TPE4N in amorphous states, are shown in Fig. S7–S11 in the ESI.† The emission bands also somewhat increased with decreased temperature due to a restriction of molecular vibiration,^16^ but in contrast to HOFTPE4N no drastic emission increment or “turn-on” process is observed. Therefore, the intramolecular rotations of nitro-substituted phenyls in the α pores explain the emission quenching in HOFTPE4N, which is consistent with the nature of AIE phenomena. With the destruction of α pores, the space for intramolecular rotations is blocked, and emission is turned on. The emission bands of HOFTPE3N and TPE3N in the amorphous state are significantly blue-shifted when the temperature decreased as shown in Fig. S9 and S10.† This is mainly attributed to the rotation and vibration of the excited state being reduced which enlarges the energy gap of the π–π* transitions.

## Conclusions

In summary, HOFs based on the building blocks TPE3N and TPE4N have provided new insights into the nature of AIE phenomena and have led to a kind of supramolecular mechanoluminescent material. Single-crystal X-ray diffraction analysis reveals that the HOFTPE3N supramolecular structure contains pores with a size of 7.655 Å × 7.655 Å, whereas the HOFTPE4N structure contains two types of pores with sizes of 5.855 Å × 5.855 Å (α pores) and 7.218 Å × 7.218 Å (β pores). Furthermore, turn-on mechanoluminescent properties are observed specifically for HOFTPE4N. The mechanism of the emission turn-on process for HOFTPE4N is supported by pXRD, DSC and temperature-dependent emission studies. Emission quenching in HOFTPE4N is attributed to the α pores, which provide sufficient space for the intramolecular rotations of the internal nitro-substituted phenyl groups. We have demonstrated that emission can also be controlled (turned on/turned off) by breaking or reforming structures similar to the α pores, or the entire HOF supramolecular structure. This study has established that a hydrogen-bonded organic framework is a unique structural environment demonstrating that intramolecular rotations are a key feature of AIE and thus this presents a new strategy for designing functional materials with mechanoluminescent properties.
