# Supplementary material for: The HOF structures of nitrotetraphenylethene derivatives provide new insights into the nature of AIE and a way to design mechanoluminescent materials
Source: Chem Sci. 2016 Sep 2;8(2):1163–8. doi: 10.1039/c6sc03177c (PMC5460603; doi:10.1039/c6sc03177c)
Supplement: Supplementary file 1 [file SC-008-C6SC03177C-s001.pdf]

Supporting Information

The HOF Structures of Nitrotetraphenylethene Derivatives Provide New Insights into the Nature of AIE and a Way to Design Mechanoluminescent

Tao Yu, Depei Ou, Zhiyong Yang,* Qiuyi Huang, Zhu Mao, Junru Chen, Yi Zhang,* Siwei Liu, Jiarui Xu, Martin R. Bryce and Zhenguo Chi*

Contents

1. Experimental Details 2

2. Syntheses of TPE2N, TPE3N and TPE4N.. 2

3. X-Ray Diffraction Measurements of TPE2N, HOFTPE3N and HOFTPE4N 4

4. Selected distances in the HOFTPE4N structure 10

5. TGA spectrum of HOFTPE4N 11

6. DSC spectra of TPE2N, TPE3N and TPE4N in different states..11

7. Temperature-depended emission spectra of TPE2N, TPE3N and TPE4N in different states 13

8. ^{1}H NMR and EI mass spectra of TPE2N, TPE3N and TPE4N.. 16

9. References 19

1. Experimental Details

^{1}H NMR spectra were recorded on a Bruker AVANCE 400 spectrometer with chemical shifts recorded relative to tetramethylsilane (Me_4Si). EI mass spectra were recorded on a Thermo MAT95XP spectrometer. Elemental analyses of the compounds were performed on an Elementar Vario EL analyzer. Single-crystal X-ray data of TPE2N, HOFTPE3N and HOFTPE4N were determined on an Oxford Diffraction Germini S Ultra X-ray Single Crystal Diffractometer using a (Cu) X-ray source. Steady state fluorescence studies were performed on a Horiba Scientific Fluorolog-3 spectrofluorometer and the low-temperature emission studies were conducted with the same spectrofluorometer equipped with a Cryocon 22C temperature controller. Solid state luminescent quantum yields were measured with the Horiba Scientific Fluorolog-3 spectrofluorometer equipped with a Horiba Scientific Quanta-φ calibrated integrating sphere. PXRD experiments were performed on a Rigaku X-ray diffractometer (D/max-2200) with an X-ray source of Cu Kα (λ= 0.15406 nm) at 40 kV and 30 mA, at a scan rate of 10° (2θ) per 1 min. TGA data was recorded on a Shimadzu TGA-50 thermogravimetric analyzer at a heating rate of 20 °C/min in N_2. DSC studies were carried out on a NETZSCH DSC 204 F1 instrument under nitrogen at a heating rate of 10 °C/min.

2. Syntheses of TPE2N, TPE3N and TPE4N

The building blocks (TPE2N, TPE3N and TPE4N) were synthesized according to the synthetic procedure described below. ^{1}H NMR and EI mass spectra of TPE2N, TPE3N and TPE4N are shown in Figures S12-S17.

TPE2N: To a mixture of 95% nitric acid (5 mL) and glacial acetic acid (5 mL), tetraphenylethylene (1.00 g, 3.01 mmol) dissolved in dichloromethane (2 mL) was added. After stirring for 1.5 hours at room temperature, the solution was diluted with water, and extracted with dichloromethane for three times. The organic layer was collected and washed with water for three times. Further purification was done by column chromatography on silica gel (60-230 mesh) with CH_2Cl_2-hexane (1:2, v/v) as eluent. Solvent removal yielded a light yellow solid. Yield: 0.70 g (55 %). ^{1}H NMR (400 MHz, CD_3Cl, 298 K, relative to Me_4Si): δ = 6.98–7.03 (m, 4H, $-C_6H_5$), 7.11–7.22 (m, 10H, $-C_6H_4-$ and C_6H_5), 7.98–8.01

(m, 4H, $-C_6H_4-$); High Resolution EI–MS: m/z found: 422.1256 $[M]^+$; calcd for $C_{26}H_{18}N_2O_4$: 422.1267.

TPE3N: The compound was prepared according to the preparation of **TPE2N**, except that the 95% nitric acid was changed from 5 mL to 7.5 mL and the stirring time was increased to 2 hours. Further purification was done by column chromatography on silica gel (60-230 mesh) with CH_2Cl_2-hexane (2:3, v/v) as eluent. Solvent removal yielded a yellow solid. Yield: 0.79 g (57 %). ^1H NMR (400 MHz, CD_3Cl, 298 K, relative to Me_4Si): δ = 6.95–6.99 (m, 2H, $-C_6H_5$), 7.15–7.24 (m, 9H, $-C_6H_4-$ and C_6H_5), 7.98–8.06 (m, 4H, $-C_6H_4-$); High Resolution EI–MS: m/z found: 467.1108 $[M]^+$; calcd for $C_{26}H_{17}N_3O_6$: 467.1117.

TPE4N: The compound was prepared according to the preparation of **TPE2N**, except that the 95% nitric acid was changed from 5 mL to 10 mL and the stirring time was increased to 5 hours. Further purification was done by column chromatography on silica gel (60-230 mesh) with CH_2Cl_2-hexane (1:1, v/v) as eluent. Solvent removal yielded a yellow solid. Yield: 1.12 g (73 %). ^1H NMR (400 MHz, CD_3Cl, 298 K, relative to Me_4Si): δ = 7.18 (d, 4H, 13.2Hz, $-C_6H_4-$), 8.07 (d, 4H, 13.2Hz, $-C_6H_4-$); High Resolution EI–MS: m/z found: 512.0958 $[M]^+$; calcd for $C_{26}H_{16}N_4O_8$: 512.0968.

3. X-Ray Diffraction Measurements of TPE2N, HOFTPE3N and HOFTPE4N

Single-crystal X-ray data for TPE2N, HOFTPE3N and HOFTPE4N were determined on an Oxford Diffraction Gemini S Ultra X-ray single-crystal diffractometer using graphite-monochromatized Cu-Kα radiation (λ = 1.54178 Å). The structures were solved by SHELXS-97 program and expanded using Fourier techniques. All non-H atoms of the compounds were refined with anisotropic thermal parameters. The hydrogen atoms were included in idealized positions and refined with fixed geometry with respect to their carrier atoms. The disordered solvent molecules in HOFTPE3N and HOFTPE4N were removed using SQUEEZE routine of PLATON. CCDC numbers for the single crystals of TPE2N, TPE3N and TPE4N are 1451590, 1451588 and 1451589, respectively.

Crystal data for TPE2N; $C_{26}H_{18}N_2O_4$, Formula Weight = 422.42 g/mol, monoclinic, space group P $2_1/c$, T = 293 K, Z = 4, a = 10.52308(18) Å, b = 8.79827(18) Å, c = 22.9441(4) Å, α = 90°, β = 95.4899(16)^o, γ = 90°, V = 2114.53(7) Å^3, ρ_c = 1.327 g cm^{-3}, $\mu(Cu_{K\alpha})$ =0.740 mm^{-1}, F(000) = 880. Reflections collected 7848, Independent reflections 4139 (R_{int} = 0.0148). R_1 = 0.0411 (I ＞ 2σ(I)) and wR_2 = 0.1123, GOF = 1.063.

Crystal data for HOFTPE3N; $C_{26}H_{17}N_3O_6$, Formula Weight = 467.42 g/mol, monoclinic, space group P $2_1/n$, T = 293 K, Z = 4, a = 14.7815(5) Å, b = 9.0701(3) Å, c = 21.2106(8) Å, α = 90°, β = 106.718(4)^o, γ = 90°, V = 2723.51(16) Å^3, ρ_c = 1.140 g cm^{-3}, $\mu(Cu_{K\alpha})$ = 0.689 mm^{-1}, F(000) = 968.0. Reflections collected 9150, Independent reflections 4320 (R_{int} = 0.0204). R_1 = 0.0539 (I ＞ 2σ(I)) and wR_2 = 0.1471, GOF = 1.086.

Crystal data for HOFTPE4N; $C_{26}H_{16}N_4O_8$, Formula Weight = 512.43 g/mol, tetragonal, space group P $4_2/n$, T = 173 K, Z = 8, a = 19.9155(2) Å, b = 19.9155(2) Å, c = 13.4286(2) Å, α = 90°, β = 90°, γ = 90°, V = 5326.15(11) Å^3, ρ_c = 1.278 g cm^{-3}, $\mu(Cu_{K\alpha})$ = 0.822 mm^{-1}, F(000) = 2112. Reflections collected 11147, Independent reflections 5252 (R_{int} = 0.0162). R_1 = 0.0528 (I ＞ 2σ(I)) and wR_2 = 0.1470, GOF = 1.060.

Table S1. Bond distances (Å) for TPE2N

**Atom**	**Atom**	**Length/Å**	**Atom**	**Atom**	**Length/Å**
C2	C3	1.3788(19)	C17	C18	1.378(2)
C2	C1	1.394(2)	C4	C3	1.381(2)
C8	C7	1.3603(18)	C4	N30	1.4715(17)
C8	C21	1.4882(16)	C18	C19	1.381(2)
C8	C9	1.4935(17)	C18	N27	1.4688(18)
C1	C6	1.3955(18)	C15	C20	1.4003(19)
C1	C7	1.4937(17)	C19	C20	1.380(2)
C9	C10	1.3936(19)	O29	N27	1.2227(18)
C9	C14	1.3940(19)	O32	N30	1.216(2)
C7	C15	1.4865(18)	N27	O28	1.2255(18)
C5	C4	1.378(2)	N30	O31	1.224(2)
C5	C6	1.3863(19)	C10	C11	1.387(2)
C21	C26	1.396(2)	C13	C12	1.380(2)
C21	C22	1.396(2)	C25	C24	1.376(3)
C16	C17	1.386(2)	C22	C23	1.384(2)
C16	C15	1.3950(18)	C11	C12	1.380(2)
C14	C13	1.381(2)	C24	C23	1.380(3)
C26	C25	1.382(2)			

Table S2. Bond angles for TPE2N

**Atom**	**Atom**	**Atom**	**Angle/°**	**Atom**	**Atom**	**Atom**	**Angle/°**
C3	C2	C1	121.44(13)	C3	C4	N30	118.22(14)
C7	C8	C21	124.16(11)	C17	C18	C19	122.14(13)
C7	C8	C9	121.37(11)	C17	C18	N27	119.32(13)
C21	C8	C9	114.46(10)	C19	C18	N27	118.50(13)
C2	C1	C6	118.49(12)	C16	C15	C20	118.25(12)
C2	C1	C7	119.66(11)	C16	C15	C7	121.17(12)
C6	C1	C7	121.84(12)	C20	C15	C7	120.56(11)
C10	C9	C14	118.46(12)	C20	C19	C18	118.56(13)
C10	C9	C8	121.42(11)	C19	C20	C15	121.24(13)
C14	C9	C8	119.99(12)	C2	C3	C4	118.24(14)
C8	C7	C15	123.46(11)	O29	N27	O28	123.15(14)
C8	C7	C1	120.12(11)	O29	N27	C18	118.59(14)
C15	C7	C1	116.42(10)	O28	N27	C18	118.25(13)

C4	C5	C6	118.51(13)	O32	N30	O31	123.32(14)
C26	C21	C22	118.47(13)	O32	N30	C4	118.24(15)
C26	C21	C8	120.04(12)	O31	N30	C4	118.44(14)
C22	C21	C8	121.31(13)	C11	C10	C9	120.62(14)
C17	C16	C15	121.06(13)	C12	C13	C14	120.59(14)
C13	C14	C9	120.51(14)	C24	C25	C26	120.14(17)
C25	C26	C21	120.80(16)	C23	C22	C21	120.12(16)
C5	C6	C1	120.84(13)	C12	C11	C10	120.23(15)
C18	C17	C16	118.63(13)	C11	C12	C13	119.58(14)
C5	C4	C3	122.38(12)	C25	C24	C23	119.84(14)
C5	C4	N30	119.40(13)	C24	C23	C22	120.61(17)

Table S3. Bond distances (Å) for HOFTPE3N

Atom	Atom	Length/Å	Atom	Atom	Length/Å
C15	C20	1.394(4)	C26	C25	1.383(5)
C15	C16	1.400(4)	C26	C21	1.385(5)
C15	C7	1.486(4)	C14	C13	1.379(5)
O32	N30	1.218(6)	C2	C3	1.362(4)
C1	C6	1.393(4)	C21	C22	1.396(5)
C1	C2	1.398(4)	C22	C23	1.402(6)
C1	C7	1.500(3)	C19	C18	1.370(5)
C16	C17	1.394(4)	C17	C18	1.387(5)
C7	C8	1.354(4)	O31	N30	1.210(6)
O29	N27	1.209(7)	O34	N33	1.231(6)
C6	C5	1.399(4)	C4	C3	1.387(6)
C20	C19	1.384(4)	C4	N33	1.478(4)
C9	C10	1.389(4)	C25	C24	1.368(6)
C9	C14	1.392(5)	C13	C12	1.378(6)
C9	C8	1.498(4)	N30	C12	1.480(5)
C10	C11	1.388(5)	O28	N27	1.178(7)
C8	C21	1.486(4)	C11	C12	1.360(6)
O35	N33	1.236(6)	C24	C23	1.344(7)
C5	C4	1.359(6)	C24	N27	1.484(6)

Table S4. Bond angles for HOFTPE3N

Atom	Atom	Atom	Angle/˚	Atom	Atom	Atom	Angle/˚
C20	C15	C16	118.1(3)	C18	C19	C20	120.7(3)
C20	C15	C7	122.4(2)	C18	C17	C16	119.7(3)
C16	C15	C7	119.4(2)	C5	C4	C3	122.5(3)
C6	C1	C2	119.9(3)	C5	C4	N33	120.0(4)
C6	C1	C7	120.4(2)	C3	C4	N33	117.5(4)
C2	C1	C7	119.6(2)	C19	C18	C17	119.9(3)
C17	C16	C15	120.7(3)	C24	C25	C26	118.8(4)
C8	C7	C15	125.3(2)	C12	C13	C14	119.0(3)
C8	C7	C1	121.2(3)	O34	N33	O35	125.2(4)
C15	C7	C1	113.5(2)	O34	N33	C4	118.6(4)
C1	C6	C5	118.7(3)	O35	N33	C4	116.2(5)
C19	C20	C15	120.8(3)	O31	N30	O32	123.3(4)
C10	C9	C14	118.7(3)	O31	N30	C12	119.0(4)
C10	C9	C8	120.1(3)	O32	N30	C12	117.5(5)
C14	C9	C8	121.2(3)	C2	C3	C4	118.2(3)
C11	C10	C9	120.8(3)	C12	C11	C10	118.9(3)
C7	C8	C21	123.4(3)	C11	C12	C13	122.0(3)
C7	C8	C9	122.0(3)	C11	C12	N30	119.2(4)
C21	C8	C9	114.6(2)	C13	C12	N30	118.8(4)
C4	C5	C6	119.6(3)	C23	C24	C25	121.9(4)
C25	C26	C21	121.3(3)	C23	C24	N27	119.6(4)
C13	C14	C9	120.6(3)	C25	C24	N27	118.4(5)
C3	C2	C1	121.1(3)	O28	N27	O29	122.7(5)
C26	C21	C22	118.3(3)	O28	N27	C24	119.4(5)
C26	C21	C8	122.1(3)	O29	N27	C24	117.9(6)
C22	C21	C8	119.4(3)	C24	C23	C22	119.9(4)
C21	C22	C23	119.6(4)				

Table S5. Bond distances (Å) for HOFTPE4N

Atom	Atom	Length/Å	Atom	Atom	Length/Å
C1	C24	1.375(4)	N13	O19	1.221(3)
C1	C4	1.377(4)	C15	C28	1.392(4)
C1	N13	1.459(3)	C15	C23	1.396(4)
C2	C3	1.378(4)	C16	C20	1.387(4)
C2	C6	1.398(3)	C16	C17	1.391(4)
C3	C7	1.375(4)	C16	C21	1.490(3)
C4	C12	1.382(4)	C17	C27	1.384(4)
C5	C21	1.352(4)	C20	C25	1.389(4)
C5	C6	1.488(3)	C23	C35	1.382(4)
C5	C15	1.494(3)	C25	C32	1.379(5)
C6	C11	1.391(4)	C27	C32	1.364(5)
C7	C22	1.381(4)	C28	C31	1.388(4)
C7	N10	1.467(4)	O29	N37	1.220(5)
C8	C9	1.381(4)	C30	C31	1.355(6)
C8	C24	1.382(4)	C30	C35	1.388(6)
C9	C12	1.392(4)	C30	N33	1.486(4)
C9	C21	1.497(3)	C32	N37	1.476(4)
N10	O26	1.217(4)	N33	O34	1.201(7)
N10	O18	1.225(4)	N33	O38	1.211(7)
C11	C22	1.378(4)	O36	N37	1.207(5)
N13	O14	1.216(4)			

Table S6. Bond angles for HOFTPE4N

Atom	Atom	Atom	Angle/°	Atom	Atom	Atom	Angle/°
C24	C1	C4	122.3(2)	C23	C15	C5	119.4(3)
C24	C1	N13	118.6(2)	C20	C16	C17	119.0(2)
C4	C1	N13	119.0(2)	C20	C16	C21	121.9(2)
C3	C2	C6	120.9(2)	C17	C16	C21	119.1(2)
C7	C3	C2	118.7(2)	C27	C17	C16	120.9(3)
C1	C4	C12	118.6(2)	C16	C20	C25	120.7(3)
C21	C5	C6	123.9(2)	C5	C21	C16	123.1(2)
C21	C5	C15	122.5(2)	C5	C21	C9	122.7(2)
C6	C5	C15	113.6(2)	C16	C21	C9	114.2(2)
C11	C6	C2	118.4(2)	C11	C22	C7	118.4(2)

C11	C6	C5	119.5(2)	C35	C23	C15	120.0(3)
C2	C6	C5	122.0(2)	C1	C24	C8	118.3(3)
C3	C7	C22	122.2(2)	C32	C25	C20	118.1(3)
C3	C7	N10	117.9(2)	C32	C27	C17	118.4(3)
C22	C7	N10	119.9(2)	C31	C28	C15	120.4(3)
C9	C8	C24	121.0(2)	C31	C30	C35	122.9(3)
C8	C9	C12	119.3(2)	C31	C30	N33	119.6(4)
C8	C9	C21	119.4(2)	C35	C30	N33	117.5(4)
C12	C9	C21	121.3(2)	C30	C31	C28	118.6(3)
O26	N10	O18	123.8(3)	C27	C32	C25	122.8(3)
O26	N10	C7	118.1(3)	C27	C32	N37	118.5(3)
O18	N10	C7	118.0(3)	C25	C32	N37	118.7(3)
C22	C11	C6	121.2(2)	O34	N33	O38	123.9(4)
C4	C12	C9	120.5(2)	O34	N33	C30	118.2(5)
O14	N13	O19	122.6(3)	O38	N33	C30	117.9(5)
O14	N13	C1	118.8(2)	C23	C35	C30	118.5(3)
O19	N13	C1	118.6(3)	O36	N37	O29	124.0(3)
C28	C15	C23	119.5(3)	O36	N37	C32	118.0(3)
C28	C15	C5	120.9(3)	O29	N37	C32	118.1(3)

4. Selected distances in the HOFTPE4N structure

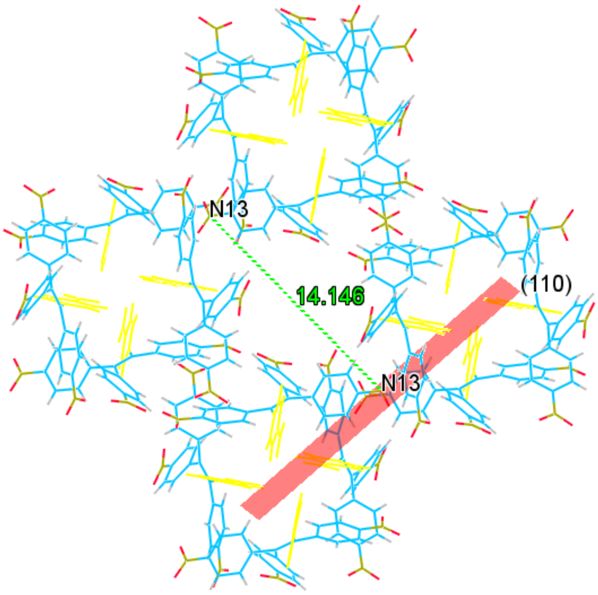

Figure S1 Distance of N13, N13 in the β type pores in the HOFTPE4N structure.

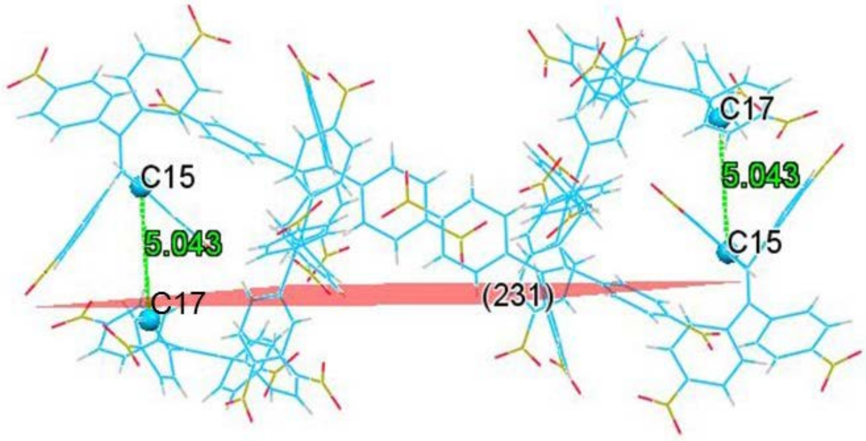

Figure S2 Distance of C15, C17 in the α type pores in the HOFTPE4N structure.

5. TGA spectrum of HOFTPE4N

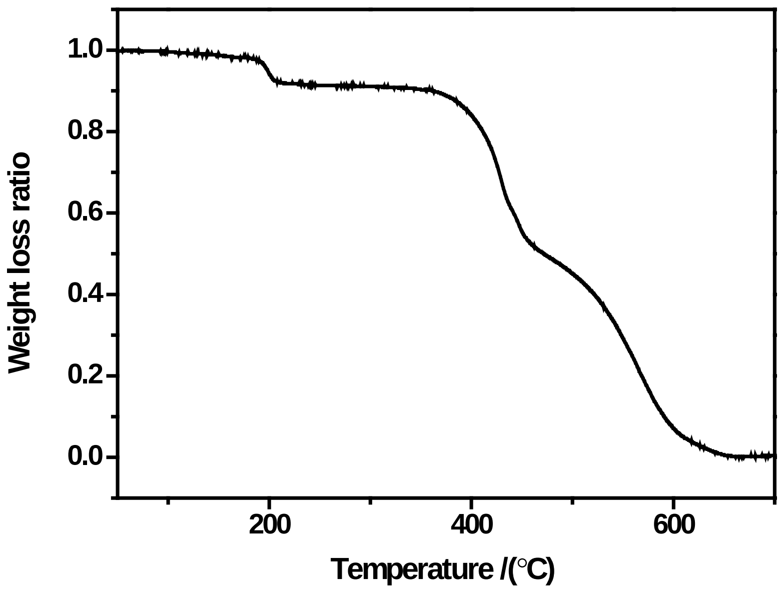

Figure S3 TGA spectrum of HOFTPE4N

6. DSC spectra of TPE2N, TPE3N and TPE4N in different states

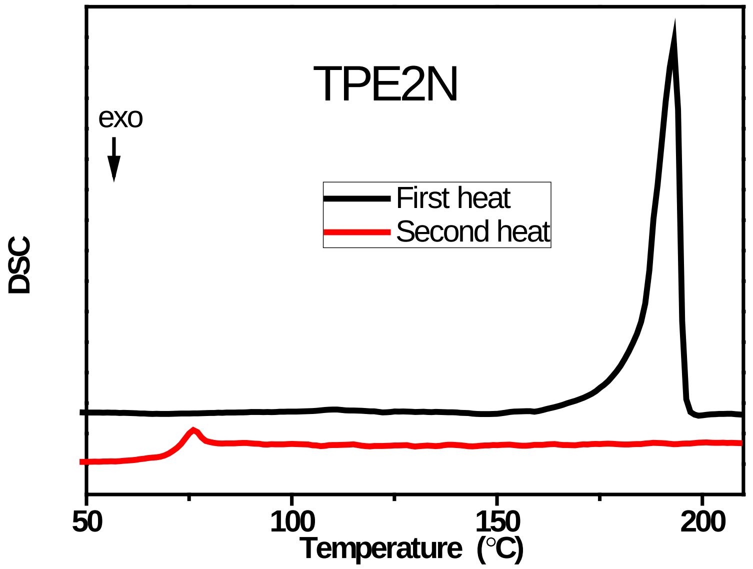

Figure S4 DSC spectra of TPE2N in the crystalline state.

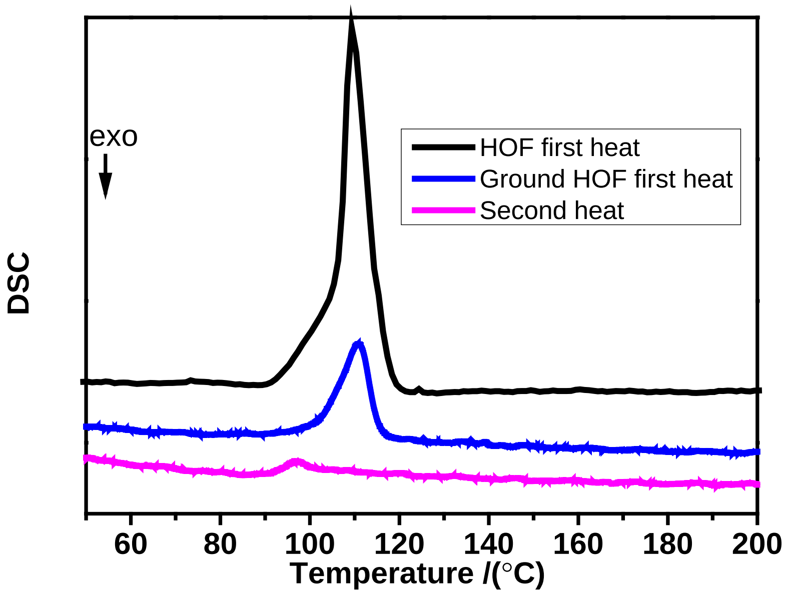

Figure S5 DSC spectra of TPE3N in different states.

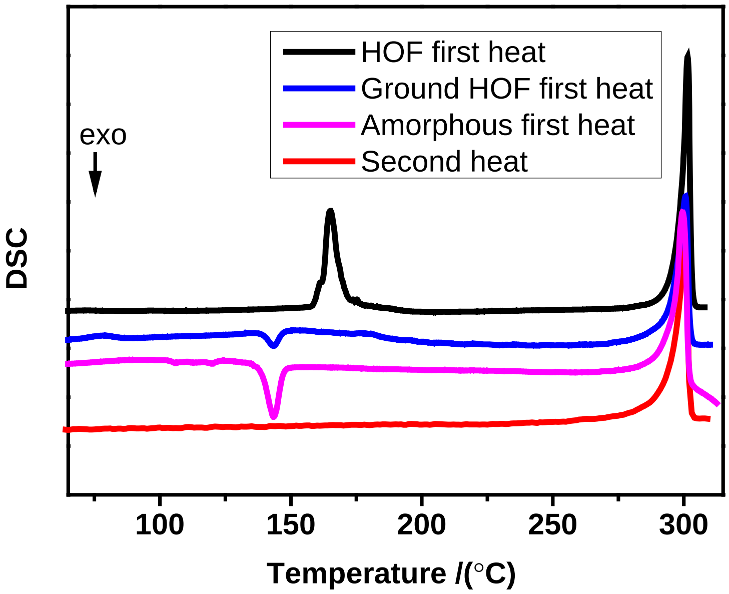

Figure S6 DSC spectra of TPE4N in different states.

7. Temperature-depended emission spectra of TPE2N, TPE3N and TPE4N in different states

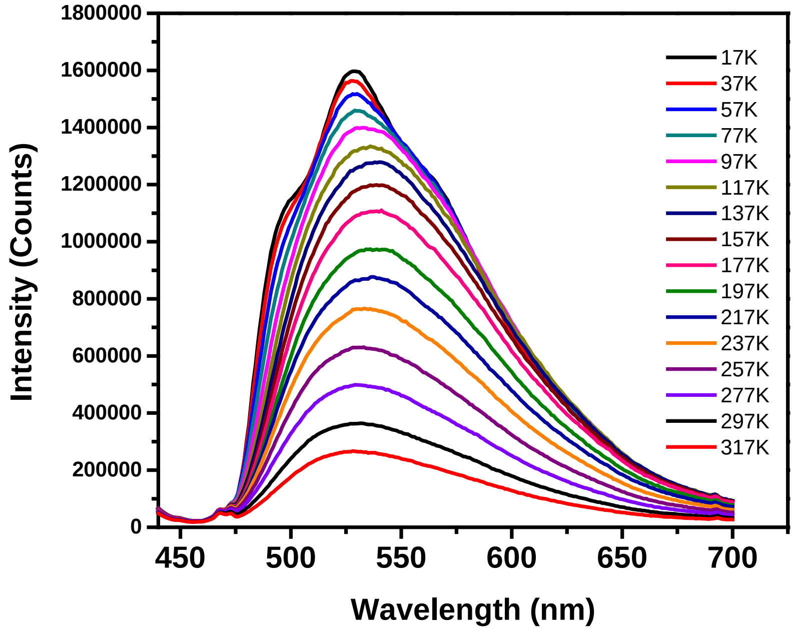

Figure S7 Temperature-depended emission spectra of TPE2N in the crystalline state.

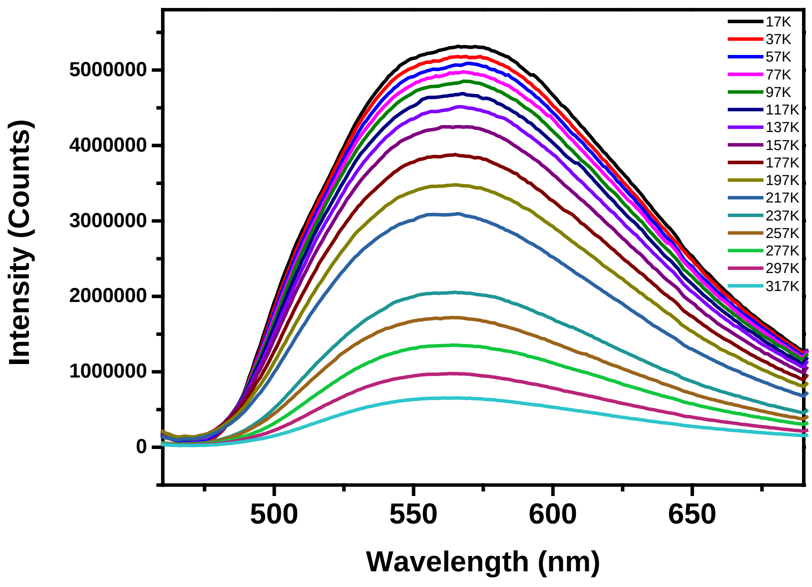

Figure S8 Temperature-depended emission spectra of amorphous TPE2N.

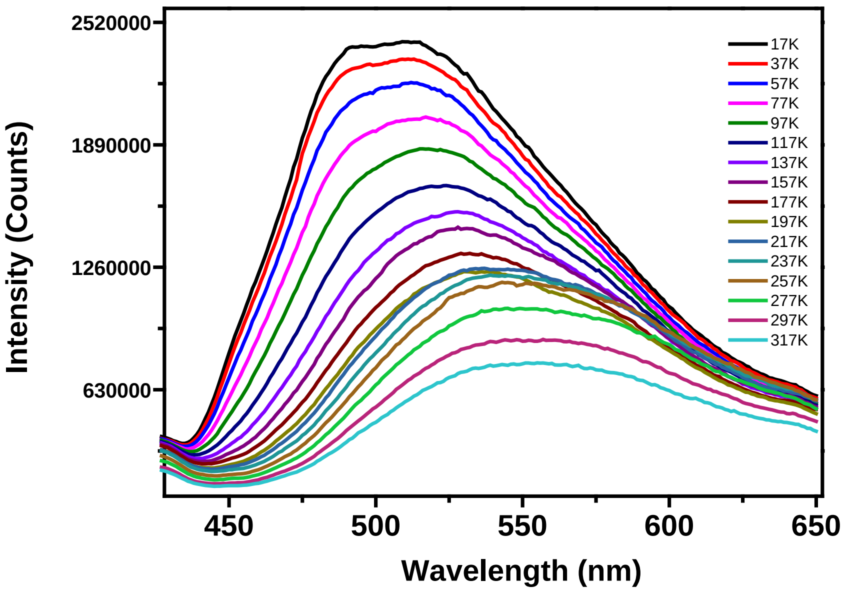

Figure S9 Temperature-depended emission spectra of HOFTPE3N.

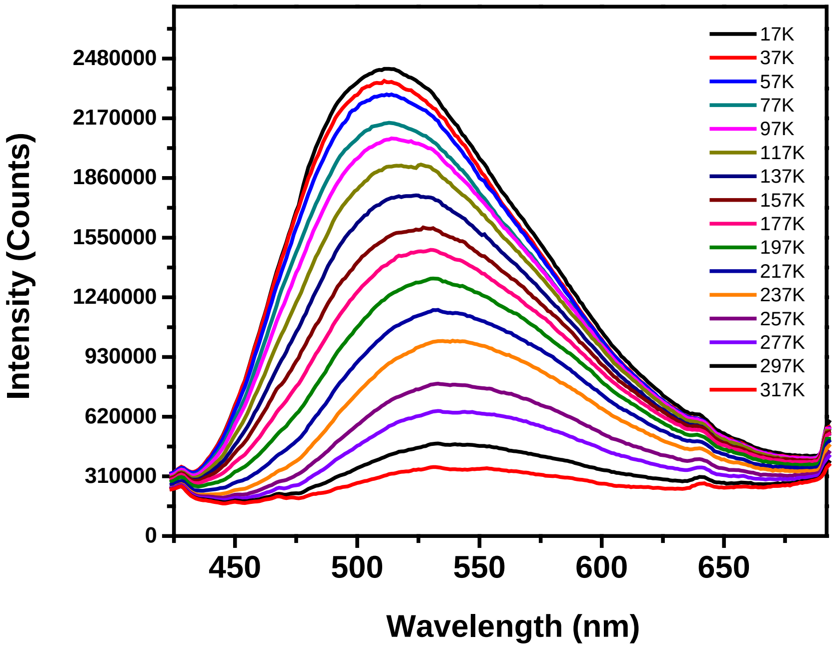

Figure S10 Temperature-depended emission spectra of amorphous TPE3N.

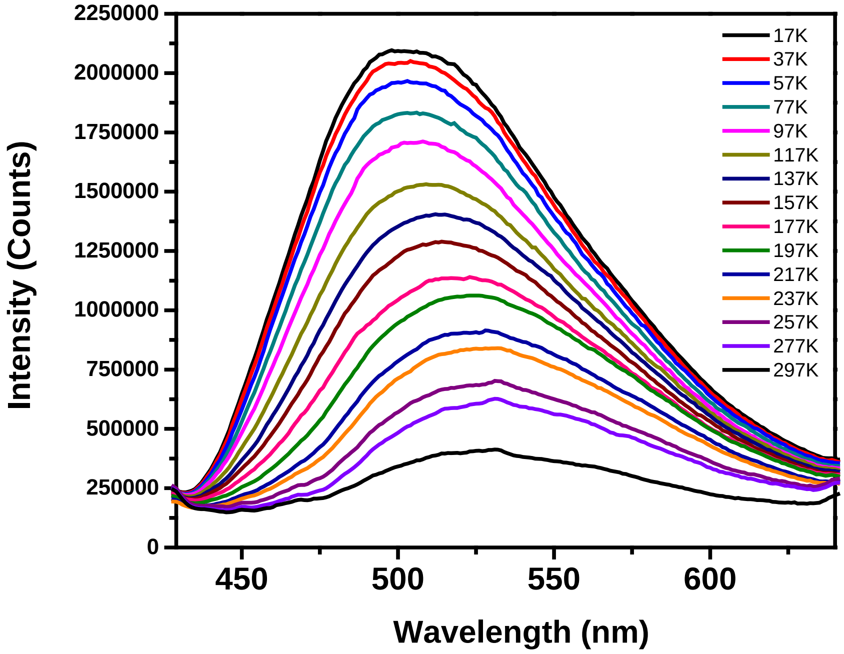

Figure S11 Temperature-depended emission spectra of amorphous TPE4N.

8. ^{1}H NMR and EI mass spectra of TPE2N, TPE3N and TPE4N

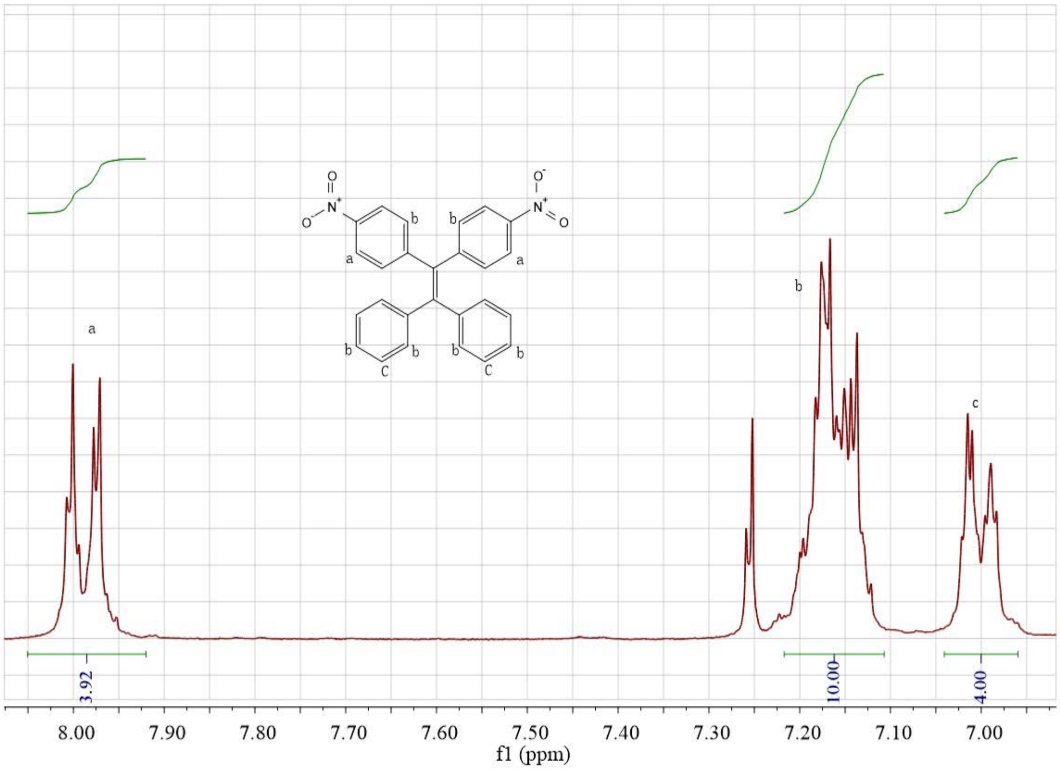

Figure S12 ^{1}H NMR spectrum of TPE2N.

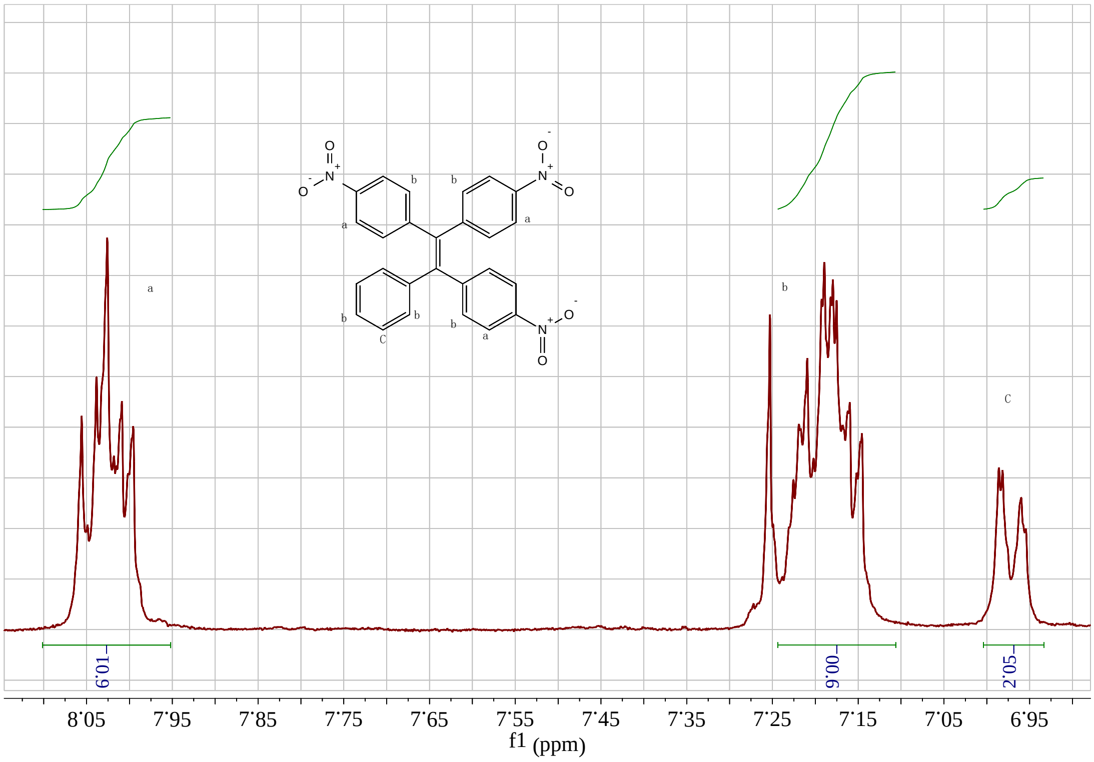

Figure S13 ^{1}H NMR spectrum of TPE3N

•

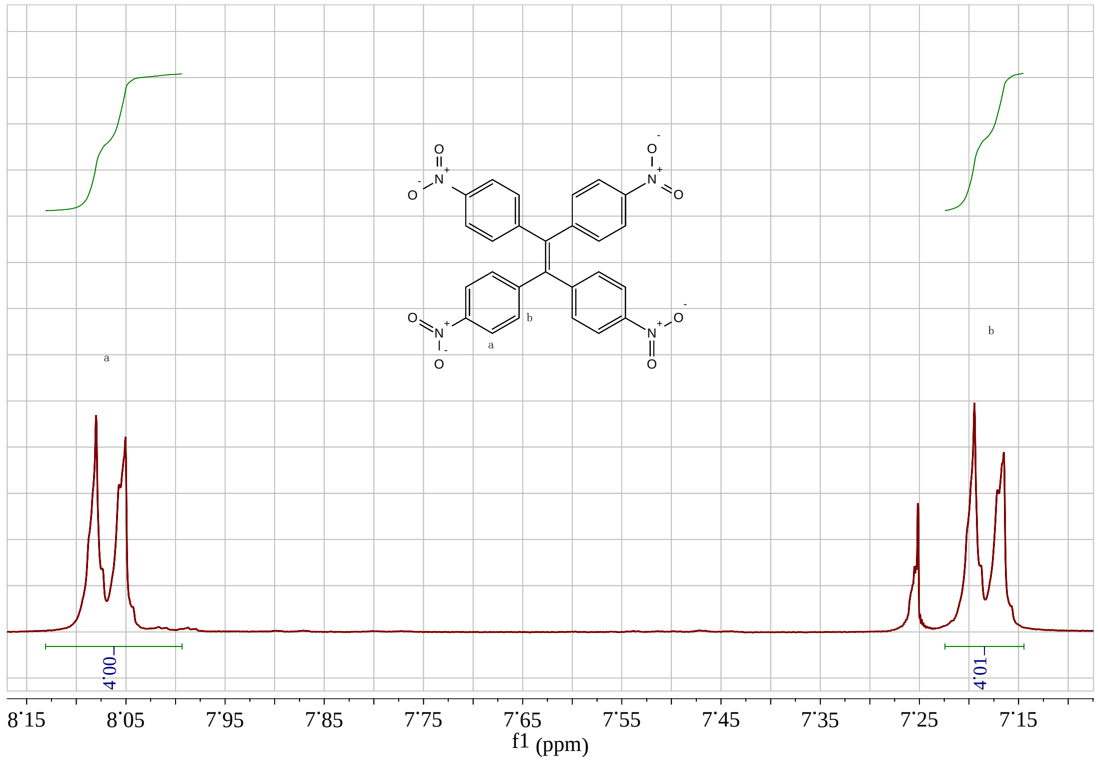

Figure S14 ^{1}H NMR spectrum of TPE4N.

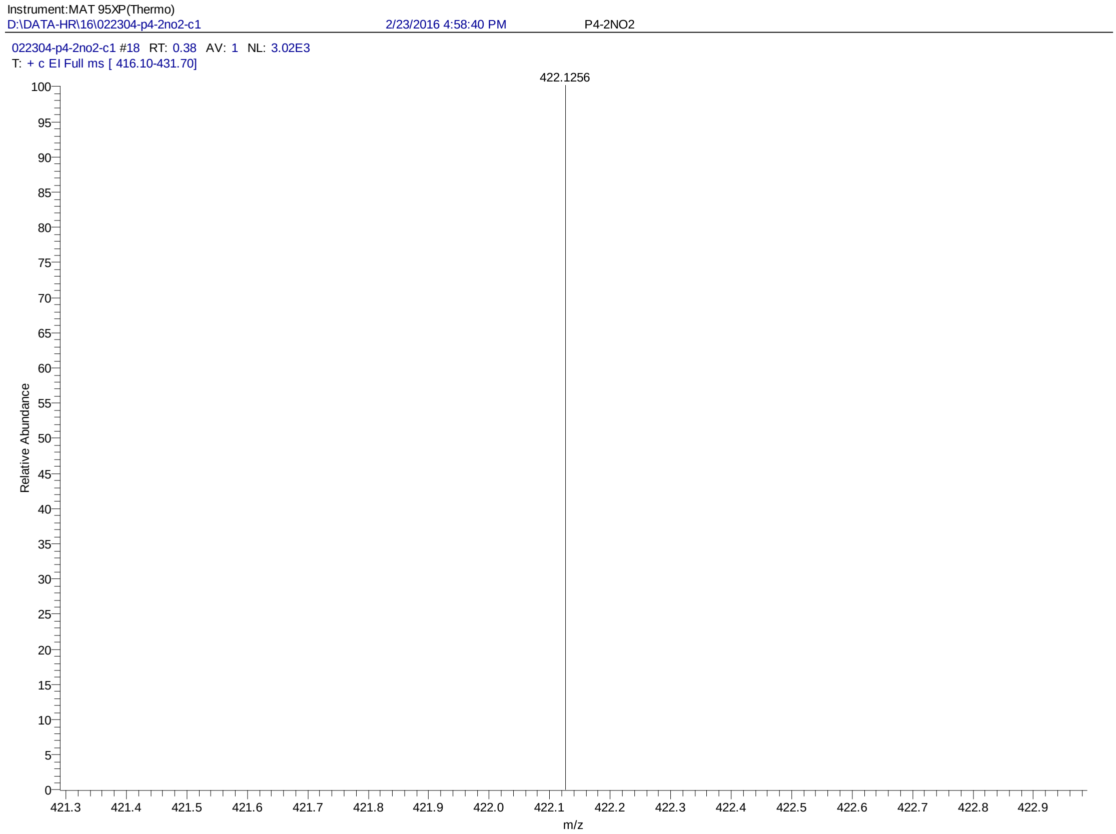

Figure S15 High resolution EI mass spectrum of TPE2N.

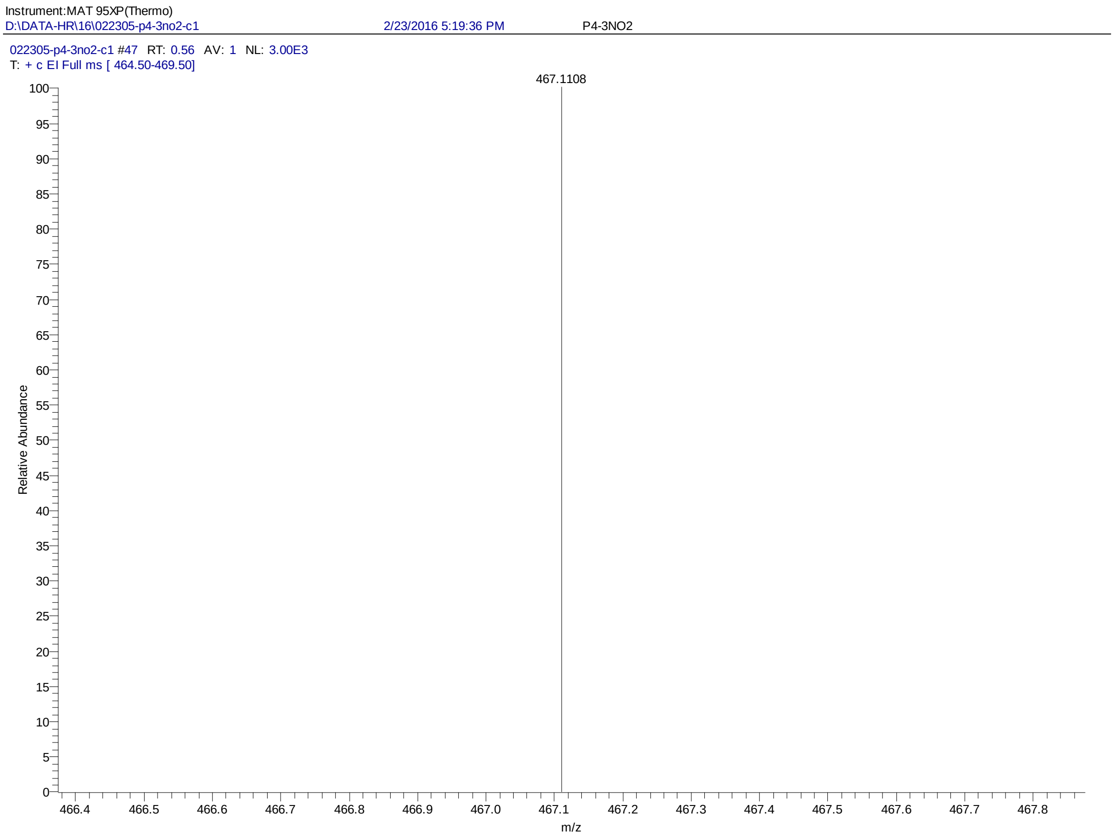

Figure S16 High resolution EI mass spectrum of TPE3N.

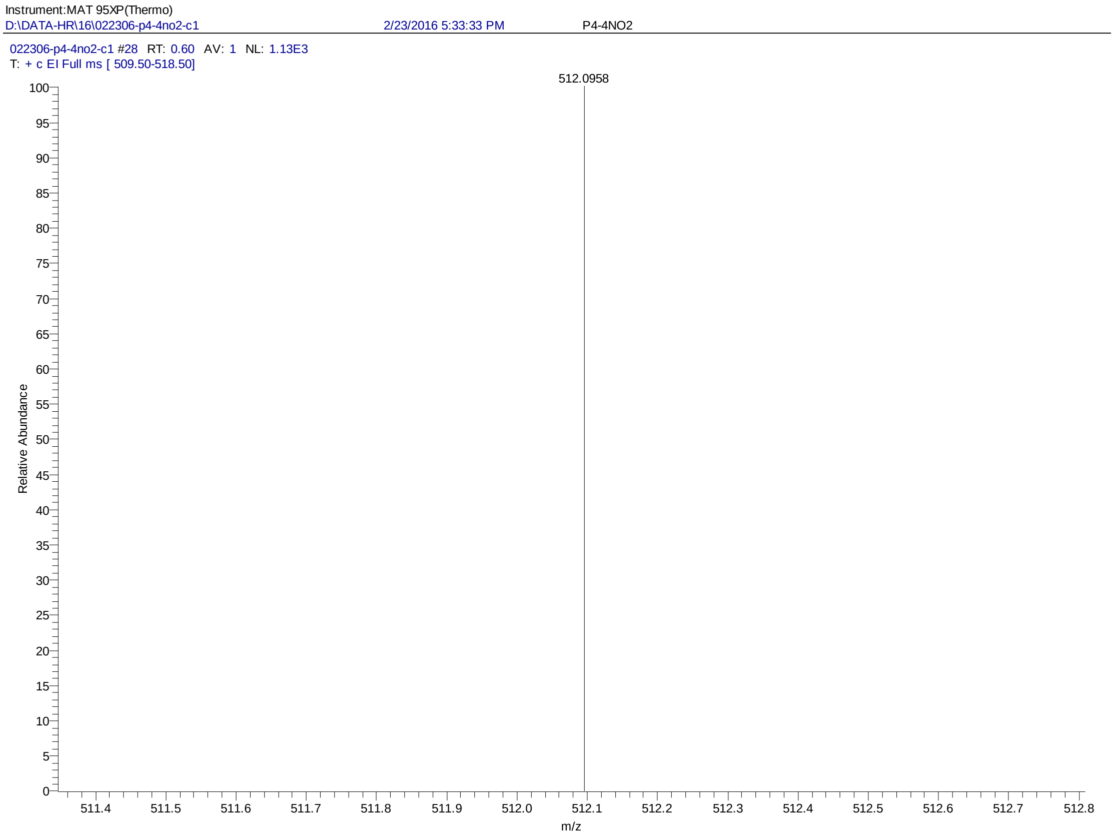

Figure S17 High resolution EI mass spectrum of TPE4N.

9. References

1. J. H. Gorvin, *J. Chem. Soc.*, **1959**, 678-682.
